# Highly efficient frequency doubling and quadrupling of a short-pulsed thulium fiber laser

**DOI:** 10.1007/s00340-018-6925-x

**Published:** 2018-03-22

**Authors:** Lin Xu, Sijing Liang, Qiang Fu, David P. Shepherd, David J. Richardson, Shaiful Alam

**Affiliations:** Optoelectronics Research Centre, University of Southampton, Southampton, SO17 1BJ UK

## Abstract

We report the second harmonic generation and fourth harmonic generation of the output from a short-pulsed (~ 80 ps) thulium-doped fiber laser, generating 976 and 488 nm wavelengths with high efficiency. With a narrow-linewidth (0.5 nm) pump at a power of 3.2 W, a second harmonic power of 2.4 W was generated at 976 nm with a conversion efficiency reaching 75%. For FHG, 690 mW of power at 488 nm was obtained from frequency doubling of 976 nm with a conversion efficiency of 30%.

## Introduction

Thulium (Tm) doped fiber lasers and amplifiers have generated great interest for a wide range of applications, such as gas sensing, material processing and environmental monitoring, due to the broad gain bandwidth of Tm at around 2 µm [1, 2]. A number of demonstrations have shown that Tm-doped fiber lasers and amplifiers can show gain extending from 1660 to 2050 nm [3–5]. As a result of this broad emission band, such systems are suitable to generate wavelengths in the range of 830 – 1025 nm through second harmonic generation (SHG), which includes some wavelengths between 900 and 980 nm that traditional solid-state lasers find hard to achieve. Short-pulsed 9xx-nm lasers have many attractive applications including medical instrumentation, spectroscopy, remote sensing, and free-space communication [6, 7]. Solutions based on semiconductor devices have been proposed with electrically modulated distributed Bragg reflectors but can only generate Watt-level peak powers [8]. Among other solutions, ytterbium (Yb) doped fibers present high emission cross-section around 976 – 980 nm when pumped around 915 nm [9]. However, gain competition between the pure three-level transition and quasi-four-level laser operation at longer wavelengths is a major problem for operation on this zero-phonon line. To overcome this difficulty, fibers with non-standard geometries need to be carefully designed [9, 10]. Furthermore, Yb-doped fiber lasers cannot be operated at wavelengths much below 970 nm.

It has been demonstrated that frequency doubling of Tm-doped fiber lasers can generate 950 nm light with a 60% conversion efficiency in the nanosecond (ns) operation regime [11]. A narrow-linewidth continuous-wave Tm-doped fiber laser has also been demonstrated giving frequency-doubled output at 970 nm with a conversion efficiency of 32.7% [12]. In this paper, we investigate the frequency doubling of a picosecond (ps) Tm-doped fiber laser and demonstrate a conversion efficiency as high as 75% which is, to the best of our knowledge, the highest efficiency yet for a frequency-doubled Tm-doped fiber laser. Furthermore, we explored the frequency quadrupling of the ps Tm-doped fiber laser to generate blue light for potential applications such as data storage, displays and spectroscopy [13]. An output power of 690 mW is obtained at a wavelength of 488 nm.

## Experimental setup and results

### Tm-doped fiber laser system

**Fig. 1:**
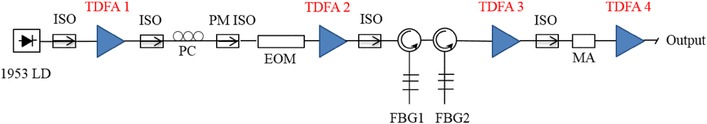
Schematic of the MOPA system. *1953 LD* laser diode at 1953 nm, *ISO* isolator, *TDFA* thulium doped fiber amplifier, *PC* polarization controller, *PM ISO* polarization maintaining isolator, *EOM* electro-optic modulator, *FBG* fiber Bragg grating, *MA* mode adaptor

Figure 1 shows the schematic of the Tm-doped fiber master oscillator power-amplifier (MOPA) system. A low-power 1953-nm InGaAs/InP Fabry–Perot diode was used as a seed for the MOPA, which was gain-switched by electrical pulses at a 1-MHz repetition rate. The pulsed seed laser, with ~ 100-ps pulse duration, had a spectral bandwidth of 0.9 nm and an average power of ~ 1 µW. A Tm-doped single-mode fiber (OFS, TmDF200) with a core diameter of ~ 4 µm, which was core-pumped by an in-house-built Er/Yb co-doped fiber laser (1.5 W at maximum) at 1565 nm, was employed as the first preamplifier providing about 40 dB of gain for the seed pulses. The excess amplified spontaneous emission (ASE) generated from the first preamplifier was eliminated by an electro-optic modulator (EOM), which acted as a time gate to pass only the optical pulses. In spite of the 9.7-dB insertion loss of the EOM and associated polarization-sensitive components, the signal after the EOM had an average power of 1.2 mW with a good optical signal-to-noise ratio (OSNR) of ~ 25 dB. A second 1565-nm core-pumped Tm-doped fiber amplifier, compromised of in-house fabricated single-mode Tm-doped fiber and Er/Yb co-doped fiber laser, increased the signal power to 48 mW. The detailed design consideration of the Tm-doped fiber amplifier system can be found in our previous work [14].

**Fig. 2:**
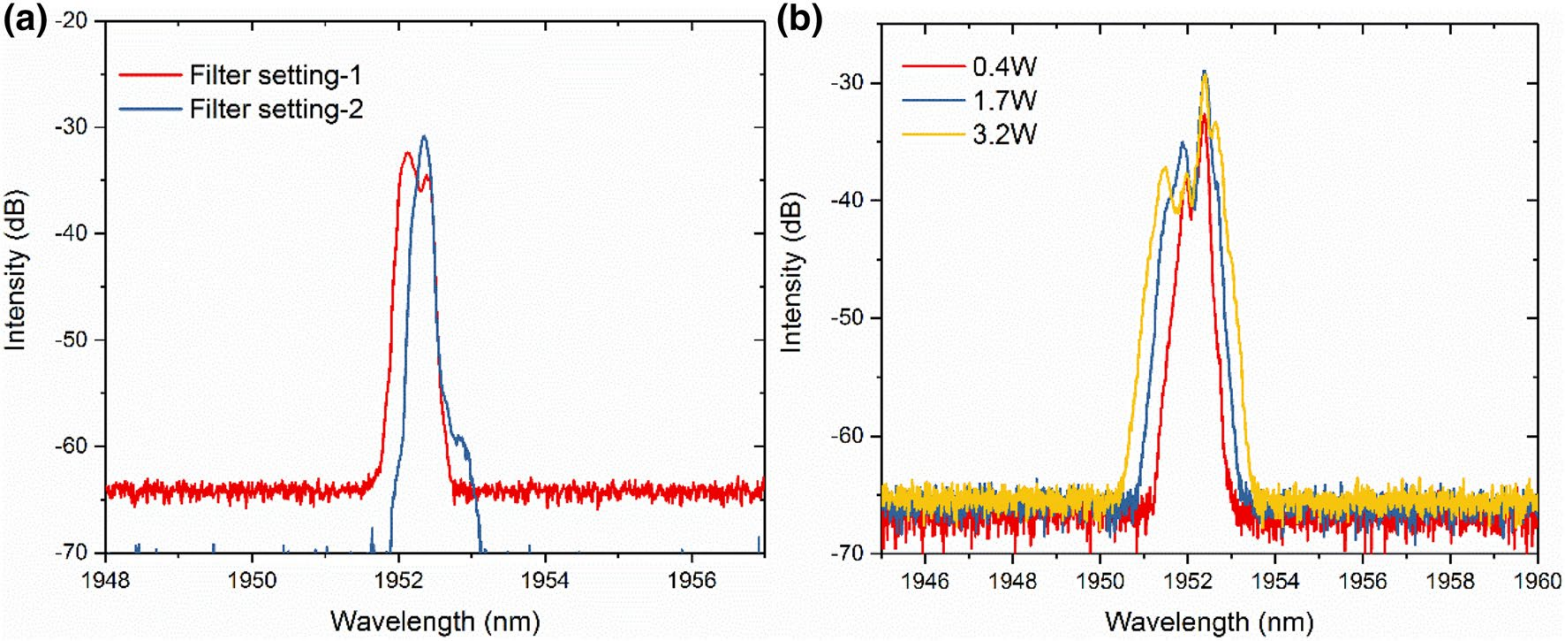
**a** Signal spectrum measured after the grating-based filter; **b** spectrum measured after the polarization-sensitive isolator with different power

A grating-based filter, comprising a pair of circulators and two fiber Bragg gratings (FBGs), was used after the second amplifier to remove further ASE and narrow the bandwidth of the signal spectrum, which was broadened due to the nonlinear effects of self-phase modulation (SPM) and modulation instability (MI). With the FBGs set for achieving maximum power, the transmitted signal had a spectrum with a 3-dB bandwidth of 0.3 nm, but with a double-peak structure, as shown with caption of ‘Filter setting-1’ in Fig. 2a. The grating-based filter also improved the OSNR of the signal spectrum to > 30 dB. To compensate the high loss induced by the grating-based filter and avoid causing additional nonlinearities, a cladding-pumped third amplifier stage was built with a 2.5-m-long in-house-fabricated Tm-doped fiber with a core diameter of 11 µm. The third amplifier stage, pumped by 2 W at 790 nm, boosted the signal power from 3 mW after the filter to 60 mW and the resulting signal was coupled into the final stage amplifier. This amplifier comprised a large-mode-area Tm-doped fiber (Nufern, LMA-TDF-25P/250-HE) having a core/cladding diameter of 25 µm/250 µm and NA of 0.09/0.46, with a length of 1.3 m. This stage was pumped by two spatially combined 790-nm laser diodes with a maximum output power of 60 W. An in-house-fabricated mode-field adaptor was used before the final-stage amplifier to match the fundamental mode of the two dissimilar fibers and hence reduce the coupling loss and improve the beam quality. The output from the final-stage amplifier was measured to have an average power of 6 W before any spectral side lobes were observed due to MI. The output pulse, which was temporally compressed along the amplifier chain as a result of the interplay between dispersion and SPM, was measured with an autocorrelator (APE Pulsecheck) and had a duration of 35 ps. The output beam was measured to have a beam quality of *M*^2^ ~ 1.3.

### Frequency doubling

**Fig. 3:**
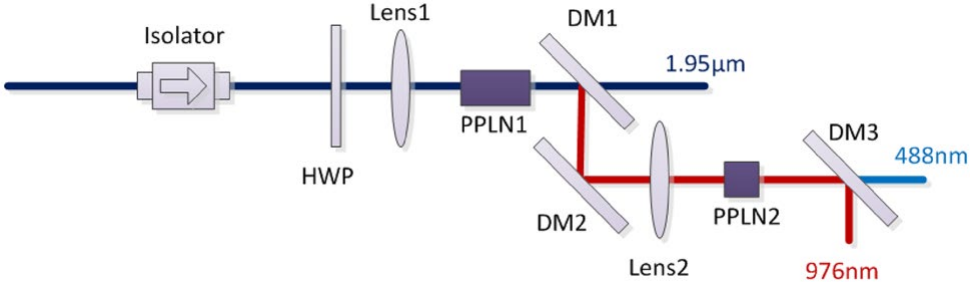
Experimental setup of frequency doubling and quadrupling

To prevent any feedback into the amplifier system and to define a well-defined linear state of polarization for subsequent frequency conversion, the output beam from the MOPA was passed through a polarization-sensitive isolator. The MOPA itself did not use polarization-maintaining fibers and components after the EOM and thus there was some transmission loss for the isolator due to depolarization. Consequently, a reduced maximum average power of 3.2 W was available after the isolator for the SHG. The spectrum for different powers measured after the isolator can be found in Fig. 2b. A half-wave plate was used after the isolator to rotate the linear polarization to realize phase matching for the SHG, as shown in Fig. 3. The beam after the half-wave plate was focused into a periodically poled 5% MgO-doped LiNbO_3_ (PPLN) crystal by a focal lens with a beam waist of 200 µm (1/*e*^2^ radius of intensity). The focusing beam size was experimentally chosen as a compromise between achieving the highest possible efficiency and avoiding beam quality degradation due to back-conversion. The PPLN crystal (Covesion, Ltd.), which had a length of 20 mm and grating periods from 28.4 to 33.2 µm, was mounted in an oven to allow temperature tuning in a range from 20 to 200 °C with a precision of 0.1 °C. Both end facets of the PPLN were anti-reflection (AR, *R* < 1%) coated at the fundamental (1952 nm) and second harmonic (976 nm) wavelengths. A dichroic mirror (DM1 in Fig. 3), with a high transmission at 1952 nm and high reflectivity at the 976 nm, was used to separate the second harmonic beam from the fundamental.

**Fig. 4:**
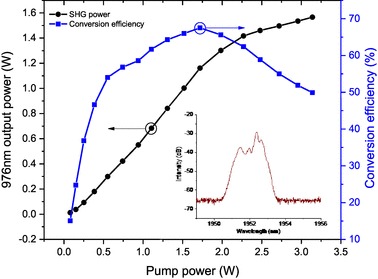
Experimental results of frequency doubling. (Left) 976 nm output power as a function of pump power. (Right) Power conversion efficiency versus pump power. Inset shows the pump spectrum at 3.2 W with filter setting-1

For a grating with a poling period of 29 µm, the SHG at 976 nm was observed when the PPLN crystal was heated to 100 °C. This is consistent with the value of 105 °C calculated from the Sellmeier equation for the type 0 (*ee–e* interaction) phase matching in PPLN [15]. The output power of the SHG was measured while increasing the input power from the Tm:doped fiber MOPA system. 1.6 W of 976 nm, measured after DM1, was generated at a pump power input to Lens 1 of 3.2 W, representing a single-pass power conversion efficiency of 50%. As evident in Fig. 4, the power conversion efficiency increased with the increment of input pump power and reached a maximum of 67% when the pump power was 1.7 W. However, the conversion efficiency saturated and then decreased with higher input pump power. The conversion efficiency roll-off behavior is likely to be associated with SPM-induced spectral broadening of the pump from the fiber MOPA as the output power increased, see Fig. 2b. The pump spectral bandwidth, calculated at 10 dB from the peak, contained about 90% of the power and increased from 0.5 to 1.5 nm as the output power increased from 0.5 to 3.2 W. To understand the influence of the pump spectrum on the SHG conversion efficiency, we calculated the pump acceptance bandwidth for the experiment. The Jacobi elliptic function of the SHG gain can be simplified to a sinc^2^ form in the low-conversion regime, where the pump is assumed to be un-depleted [16]. As a result, the pump acceptance bandwidth, defined by the bandwidth at which the second harmonic gain falls to one-half of the maximum, is 1.7 nm for the 20-mm-long PPLN for SHG at 1952 nm. However, it would be invalid to simplify the Jacobi elliptic function in the high-conversion regime as the width of the main lobe of the function rapidly narrows with increasing gain and thus narrows the pump acceptance [17]. From our fuller calculations, the pump acceptance bandwidth for our PPLN crystal in the large-pump-depletion regime dropped to 0.5 nm for the maximum pump intensity used in the experiment.

**Fig. 5:**
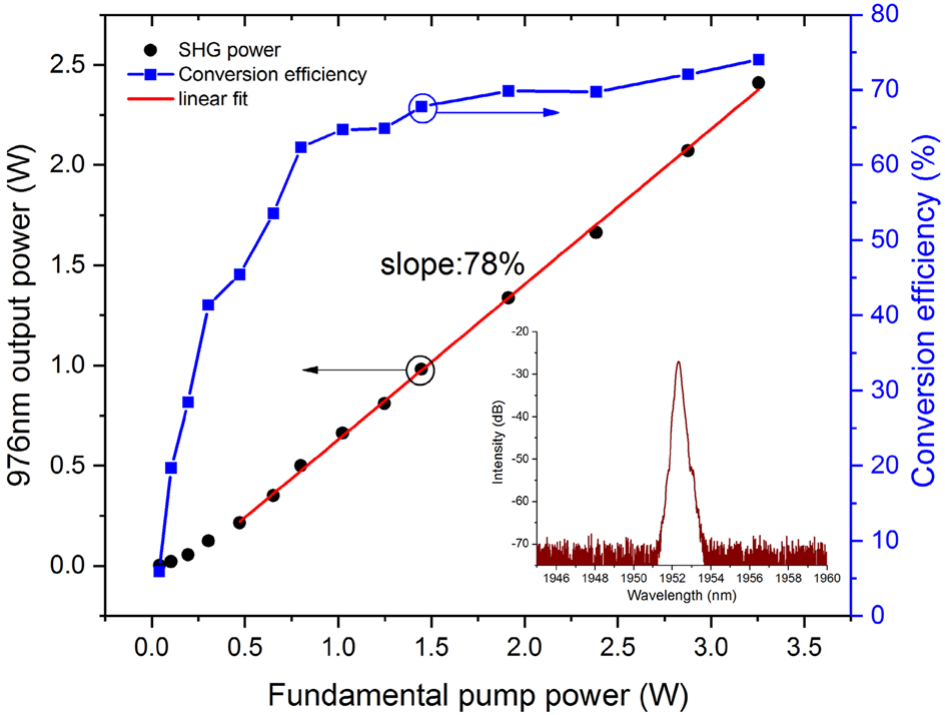
Frequency doubling results after pump spectrum was narrowed. (Left) 976 nm output power as a function of pump power. (Right) Power conversion efficiency versus pump power. Inset shows the pump spectrum at 3.2 W with filter setting-2

To improve the conversion efficiency in the high-power regime, the central wavelength of the FBG (FBG2 in Fig. 1) was shifted towards a longer wavelength by heating the FBG from room temperature (20 °C) to 60 °C, thus narrowing the combined transmission spectral window of the two FBGs. As a result the transmission bandwidth of the grating-based filter was reduced and the transmitted signal spectrum was again measured to have a 3-dB linewidth of 0.3 nm but with just a single peak, as shown with caption of ‘Filter setting-2’ in Fig. 2a. Due to the increased loss from the narrower-band filter, more pump power was required in the third pre-amplifier to achieve the 60 mW output power. After the final amplifier stage, no obvious SPM-induced spectral modulation was observed at the same output power of 3.2 W. In comparison to pre-temperature tuning of the FBG, the amplified pump spectral 10 dB-bandwidth was reduced from 1.5 to 0.5 nm, as can be seen in the inset of Fig. 5. The output pulses had a duration of ~ 80 ps, measured with a 12.5-GHz-bandwidth fast photodetector and a 50-GHz-bandwidth digital communications analyser (DCA). The increased pump pulse width originated from changes in the interplay of the filter response and the frequency chirp of the gain-switched seed pulses as the filter was tuned.

Figure 5 shows that the generated second-harmonic power increased quadratically at low pump power and linearly at high power, when measured against increasing pump power, with a slope efficiency of 78%. As expected, the conversion efficiency did not roll off when utilizing the narrow pump linewidth. A maximum power of 2.4 W at 976 nm was obtained with a pump power of 3.2 W, and the corresponding conversion efficiency reached 75%. The generated 976-nm beam had a FWHM spectral bandwidth of 0.1 nm and had a pulse width of 70 ps, as measured by a 32-GHz-bandwidth photodetector and the 50-GHz bandwidth DCA. The maximum peak power of the pump and the generated SH pulses were 45 and 34 kW, respectively.

**Fig. 6:**
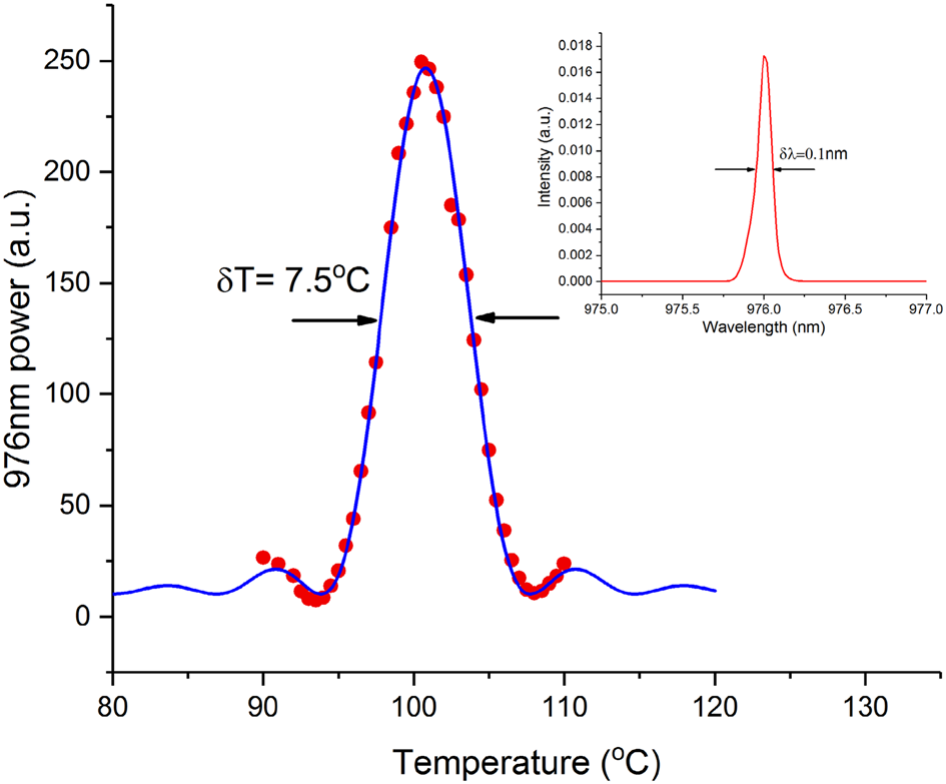
Temperature dependence of the 976 nm power (filled circles) and the sinc^2^ fit (solid curve). The inset shows the 976 nm spectrum

Characterization of the influence of the PPLN temperature on the SHG was performed by measuring the generated 976-nm power whilst tuning the crystal temperature, with the results shown in Fig. 6. The solid curve is a sinc^2^ fit to the measured data, confirming the expected temperature dependence of SHG. The FWHM of the curve is 7.5 °C, which is in good agreement with the calculated value of 8 °C from the Sellemier equation [15]. The beam quality of the 976 nm beam was measured using a scanning beam profiler, giving an *M*^2^ value of 1.7. The slightly degraded beam quality of the 976 nm beam was most likely due to some back-conversion of the SHG when operated in the high conversion efficiency regime with a peak pump intensity of 35 MW/cm^2^.

### Frequency quadrupling

**Fig. 7:**
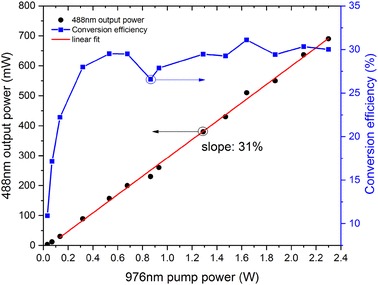
Experimental results of frequency quadrupling. (Left) 488-nm output power as a function of 976-nm pump power. (Right) Power conversion efficiency versus pump power

The frequency quadrupling stage, which produces 488 nm radiation, used a 10-mm-long PPLN crystal (Covesion, Ltd.) with poling periods from 5.17 to 5.29 µm, as shown in Fig. 3. The shorter PPLN crystal length gave a calculated acceptance bandwidth (in the low-depletion regime) of 0.14 nm in comparison to the 976-nm FWHM bandwidth of 0.1 nm. The 976-nm pump beam was focused into the PPLN crystal with a beam waist of 290 µm (1/*e*^2^ radius of intensity). Both end facets of the PPLN crystal were AR coated at the fundamental (976 nm) and second harmonic (488 nm) wavelengths. A dichroic mirror (DM3), with high transmission at the 488 nm and high reflectivity at the 976 nm, was used to extract the fourth-harmonic beam. With a PPLN phase-matching period of 5.26 µm and a temperature of 53.2 °C, frequency-quadrupled blue light at 488 nm was observed at low pump power. It was found that with increasing pump power, the operating temperature of the PPLN crystal required adjustment to optimize the 488-nm output power, which was attributed to thermal loading via visible and induced near-infrared absorption [18]. A maximum power of 690 mW at 488 nm was obtained with the PPLN set at 52.1 °C, when pumped with 2.3 W at 976 nm. As can be seen from Fig. 7, a slope efficiency of 31%, and a maximum conversion efficiency of 30% were recorded. The beam quality of the 488 nm beam was characterized and measured to be *M*^2^ ~ 2.1. The lower efficiency of the quadrupling stage is attributed to a combination of the wider-than-optimal input bandwidth, the reduced beam quality of the input, and the absorption.

## Conclusions

In conclusion, we have demonstrated efficient second harmonic generation (SHG) and fourth harmonic generation (FHG) of a short-pulsed thulium-doped fiber laser, generating 976 and 488 nm wavelengths, with good conversion efficiencies. We characterized and investigated the SHG using the same pump source with different spectral bandwidths and realized a 75% power conversion from the fundamental (1952 nm) to the SH (976 nm). A maximum power of 2.4 W at 976 nm was achieved with a pump power of 3.2 W. Blue light at 488 nm was generated (FHG) with 690 mW output power and 30% conversion efficiency. These results demonstrate that the tunability of Tm-doped fibre lasers, along with wavelength-agile efficient frequency conversion in PPLN crystals, offers a convenient route to picosecond pulses across a broad range of useful visible and near-infrared wavelengths.
